# An investigation into a semi-porous channel's forced convection of nano fluid in the presence of a magnetic field as a result of heat radiation

**DOI:** 10.1038/s41598-023-44275-4

**Published:** 2023-10-28

**Authors:** Bahram Jalili, Amirali Shateri, Ali Akgül, Abdul Bariq, Zohreh Asadi, Payam Jalili, Davood Domiri Ganji

**Affiliations:** 1 Department of Mechanical Engineering, North Tehran Branch, Islamic Azad University, Tehran, Iran; 2 Department of Computer Science and Mathematics, Lebanese American University, Beirut, Lebanon; 3 Art and Science Faculty, Department of Mathematics, Siirt University, 56100 Siirt, Turkey; 4 Department of Mathematics, Mathematics Research Center, Near East University, Near East Boulevard, 99138 Nicosia/Mersin 10, Turkey; 5 Department of Mathematics, Education Faculty, Laghman University, Mehtarlam, Laghman 2701 Afghanistan; 6 Department of Mechanical Engineering, Babol Noshirvani University of Technology, P.O. Box 484, Babol, Iran

**Keywords:** Engineering, Mathematics and computing

## Abstract

This study investigates the impact of heat radiation on magnetically-induced forced convection of nanofluid in a semi-porous channel. The research employs Akbari-Ganji's and Homotopy perturbation methods to analyze the effects of multiple parameters, including Hartmann number, Reynolds number, Eckert number, radiation parameter, and suction parameter, on the flow and heat transfer characteristics. The results demonstrate that increasing Reynolds number, suction, and radiation parameters increases temperature gradient, providing valuable insights into improving heat transfer in semi-porous channels. The study validates the proposed methods by comparing the results with those obtained from other established methods in the literature. The main focus of this work is to understand the behavior of nanofluids in semi-porous channels under the influence of magnetic fields and heat radiation, which is essential for various industrial and engineering applications. The future direction of this research includes exploring the effects of different nanoparticle shapes and materials on heat transfer performance and investigating the influence of other parameters, such as buoyancy forces and variable properties, on the flow and heat transfer characteristics. The findings of this study are expected to contribute to the development of more efficient thermal management systems in the future.

## Introduction

Forced fluid convection in a channel due to applying a magnetic field has been extensively studied over the past several decades. Recently, interest in this topic has significantly increased due to its potential applications in various fields, such as materials synthesis, bioengineering, and thermal management systems^1–3^. Magnetohydrodynamics (MHD) is a field that studies the behavior of electrically conducting fluids in the presence of a magnetic field. Hybrid nanofluids, which consist of nanoparticles dispersed in base fluids, have garnered significant attention because of their improved thermal and transport properties. This literature review aims to provide an overview of recent research on magnetohydrodynamic (MHD) hybrid nanofluid flow in various applications. Alghamdi et al.^4^ investigated the MHD hybrid nanofluid-containing medication flow through a blood artery. They studied the effects of various parameters on the flow characteristics, such as the Hartmann number, nanoparticle volume fraction, and medication concentration. The study provides insights into the behavior of hybrid nanofluids in biomedical applications. Dinarvand et al.^5^ proposed a novel hybridity model for TiO_2_–CuO/water hybrid nanofluid flow over a static/moving wedge or corner. They analyzed the effects of various parameters, such as the volume fraction of nanoparticles, the angle of the wedge, and the magnetic field's strength, on the flow and heat transfer characteristics. The study provides valuable information for the design of heat exchangers and cooling systems. Ali et al.^6^ investigated the impact of thermal radiation and non-uniform heat flux on magnetohydrodynamic (MHD) hybrid nanofluid flow along a stretching cylinder. They investigated the impact of different parameters on the flow and heat transfer characteristics, including the radiation parameter, Hartmann number, and nanoparticle volume fraction. The study provides insights into the behavior of hybrid nanofluids in industrial processes. Tlili et al.^7^ studied the 3D MHD nonlinear radiative flow of CuO–MgO/methanol hybrid nanofluid beyond an irregular dimension surface with a slip effect. They analyzed the effects of various parameters, such as nanoparticle volume fraction, radiation parameter, and slip parameter, on flow and heat transfer characteristics.

The study contributes to understanding the flow of hybrid nanofluids in complex geometries. Irfan^8^ investigated the study of Brownian motion and thermophoretic diffusion on the nonlinear mixed convection flow of Carreau nanofluid with variable properties. The study focused on the effects of Brownian motion and thermophoretic diffusion on the flow and heat transfer characteristics of the Carreau nanofluid. The findings provide insights into the behavior of nanofluids with varying properties. Irfan^9^ studied the influence of thermophoretic diffusion of nanoparticles with Joule heating in the flow of Maxwell nanofluid. The study investigated the effects of various parameters, such as the thermophoretic diffusion coefficient, Joule heating parameter, and nanoparticle volume fraction, on flow and heat transfer characteristics. The research contributes to the understanding of Maxwell nanofluid flow with thermophoretic effects.

Irfan et al.^10^ examined the aspects of Arrhenius activation energy in mixed convection Carreau nanofluid with nonlinear thermal radiation. They analyzed the effects of various parameters, such as activation energy, radiation parameter, and nanoparticle volume fraction, on flow and heat transfer characteristics. The study provides insights into the behavior of Carreau nanofluids, considering activation energy considerations. Irfan et al.^11^ conducted a theoretical analysis of the new mass flux theory and the Arrhenius activation energy in the Carreau nanofluid with magnetic influence. They investigated the effects of various parameters, such as activation energy, magnetic field strength, and nanoparticle volume fraction, on flow and heat transfer characteristics. The research contributes to understanding Carreau nanofluid flow with the mass flux theory and magnetic effects. Ahmed et al.^12^ conducted a numerical study on the unsteady flow and heat transfer of a magnetohydrodynamic (MHD) nanofluid based on carbon nanotubes (CNTs) with variable viscosity over a porous, shrinking surface. They analyzed the effects of various parameters, such as the Hartmann number, suction parameter, and nanoparticle volume fraction, on the flow and heat transfer characteristics. The study provides insights into the behavior of magnetohydrodynamic (MHD) nanofluids based on carbon nanotubes (CNTs) with varying viscosity. Ellahi^13^ investigated the effects of magnetohydrodynamics (MHD) and temperature-dependent viscosity on the flow of non-Newtonian nanofluids in a pipe and provided analytical solutions. The study investigated the impact of magnetic field strength, temperature-dependent viscosity, and nanoparticle volume fraction on the flow and heat transfer characteristics.

The findings contribute to the understanding of non-Newtonian nanofluid flow with temperature-dependent viscosity. Khan et al.^14^ studied heat transmission in the Darcy-Forchheimer flow of Sutterby nanofluid containing gyrotactic microorganisms. They analyzed the effects of various parameters on the flow and heat transfer characteristics, such as the Darcy-Forchheimer parameter, the gyrotactic microorganism parameter, and the nanoparticle volume fraction. The research provides insights into the behavior of Sutterby nanofluids containing gyrotactic microorganisms. Zeeshan et al.^15^ investigated the hydromagnetic flow of two immiscible nanofluids under the combined effects of Ohmic and viscous dissipation between two parallel moving plates. They investigated the impact of different factors, including magnetic field strength, Ohmic dissipation parameter, and nanoparticle volume fraction, on the flow and heat transfer characteristics. The study contributes to the understanding of immiscible nanofluid flow, including Ohmic and viscous dissipation effects.

Sheremet et al.^16^ investigated the effect of a magnetic field on the unsteady natural convection in a cavity with wavy walls filled with a nanofluid using Buongiorno's mathematical model. They analyzed the effects of different parameters, including Hartmann number, nanoparticle volume fraction, and Rayleigh number, on the flow and heat transfer characteristics. The study provides insights into the behavior of nanofluids in cavities with wavy walls under the influence of a magnetic field. Mehryan et al.^17^ studied the melting behavior of phase change materials in a non-uniform magnetic field caused by two variable magnetic sources. They analyzed the effects of various parameters, such as magnetic field strength, nanoparticle volume fraction, and melting temperature, on the melting process. The study contributes to understanding phase change materials and their behavior under non-uniform magnetic fields. Dogonchi et al.^18^ investigated the free convection of a copper–water nanofluid in a porous gap between a hot rectangular cylinder and a cold circular cylinder under the influence of an inclined magnetic field. They analyzed the effects of various parameters, such as the Hartmann number, nanoparticle volume fraction, and Darcy number, on flow and heat transfer characteristics. The study provides insights into the behavior of nanofluids in porous media under the influence of an inclined magnetic field. Heysiattalab et al.^19^ investigated the anisotropic behavior of magnetic nanofluids (MNFs) during filmwise condensation on a vertical plate under a uniform variable-directional magnetic field. They analyzed the effects of various parameters, such as magnetic field strength, nanoparticle volume fraction, and condensation rate, on the flow and heat transfer characteristics. The study contributes to understanding magnetic nanofluids and their anisotropic behavior during filmwise condensation. Heidary et al.^20^ conducted a numerical study on the effect of a magnetic field on the forced convection of nanofluid in a channel. They investigated the effects of various parameters, such as the Hartmann number, nanoparticle volume fraction, and Reynolds number, on flow and heat transfer characteristics. The research provides insights into nanofluids' behavior in channels under a magnetic field's influence. Selimefendigil et al.^21^ studied the phenomenon of conjugate natural convection in a cavity with a conductive partition. The cavity was filled with different nanofluids on each side of the partition. They analyzed the effects of various parameters, such as the Rayleigh number, nanoparticle volume fraction, and partition thickness, on the flow and heat transfer characteristics. The study provides insights into the behavior of nanofluids in cavities with conductive partitions. Chamkha et al.^22^ investigated the entropy generation and natural convection of a CuO-water nanofluid in a C-shaped cavity under the influence of a magnetic field. They analyzed the effects of various parameters, such as the Hartmann number, nanoparticle volume fraction, and Rayleigh number, on flow and heat transfer characteristics. The study contributes to understanding entropy generation and natural convection of nanofluids in C-shaped cavities under the influence of magnetic fields.

Mehrez et al.^23^ studied the enhancement of heat exchange in the flow of ferrofluid into a rectangular channel in the presence of a magnetic field. They analyzed the effects of various parameters, such as magnetic field strength, nanoparticle volume fraction, and Reynolds number, on the flow and heat transfer characteristics. The research provides insights into the behavior of ferrofluids in rectangular channels under the influence of a magnetic field. Jalili et al.^24^ investigated the effect of magnetic and boundary parameters on the analysis of flow characteristics in a micropolar ferrofluid through a shrinking sheet with effective thermal conductivity. They analyzed the effects of various parameters, such as the Hartmann number, shrinking parameter, and nanoparticle volume fraction, on the flow and heat transfer characteristics. The study contributes to understanding the flow of micropolar ferrofluid through shrinking sheets, taking into account magnetic and boundary effects. Jalili et al.^25^ conducted a thermal analysis of Williamson fluid flow with Lorentz force on a stretching plate. They investigated the effects of various parameters, such as magnetic field strength, nanoparticle volume fraction, and stretching parameter, on the flow and heat transfer characteristics. The research provides insights into the behavior of Williamson fluid flow under the influence of Lorentz force. Jalili et al.^26^ conducted a numerical analysis of magnetohydrodynamic (MHD) nanofluid flow and heat transfer in a circular porous medium containing a Cassini oval, considering the effects of Lorentz and buoyancy forces. They investigated the impact of different parameters, including magnetic field strength, buoyancy parameter, and nanoparticle volume fraction, on the flow and heat transfer characteristics. The study contributes to understanding magnetohydrodynamic (MHD) nanofluid flow in circular porous media with Cassini ovals. Jalili et al.^27^ conducted a thermal analysis of Williamson fluid flow with Lorentz force on a stretching plate. They investigated the effects of various parameters, such as magnetic field strength, nanoparticle volume fraction, and stretching parameter, on the flow and heat transfer characteristics. The research provides insights into the behavior of Williamson fluid flow under the influence of the Lorentz force. Analytical methods have been employed to study various aspects of magnetohydrodynamic (MHD) hybrid nanofluid flow. Researchers have favored the homotopy perturbation method (HPM) as an analytical technique to solve a range of heat transport and fluid mechanics differential equations in recent studies^28,29^. The AkbariGanji method (AGM), another analytical approach, has been introduced to address certain issues in fluid thermal analysis^30,31^.

The literature review has shown that the flow of MHD hybrid nanofluids has been extensively studied in various applications, including biomedical, industrial, and heat transfer systems. However, there is still a research gap in understanding the behavior of nanofluids in semi-porous channels under the influence of magnetic fields and heat radiation. The present study, titled "Investigating the Impact of Heat Radiation on Magnetically-Induced Forced Convection of Nanofluid in a Semi-Porous Channel", aims to fill this gap by examining how heat radiation affects magnetically-induced forced convection of CuO-water nanofluid in a semi-porous channel. The study utilizes Akbari-Ganji's and the Homotopy perturbation methods, both advantageous analytical techniques for solving complex thermal problems. The research provides valuable insights into improving heat transfer in semi-porous channels by analyzing the effects of multiple parameters, such as the Hartmann number, Reynolds number, Eckert number, radiation parameter, and suction parameter. This is essential for various industrial and engineering applications. The results of this study are expected to contribute to the development of more efficient thermal management systems in the future.

## Mathematical framework

### Governing formulas

**Figure 1:**
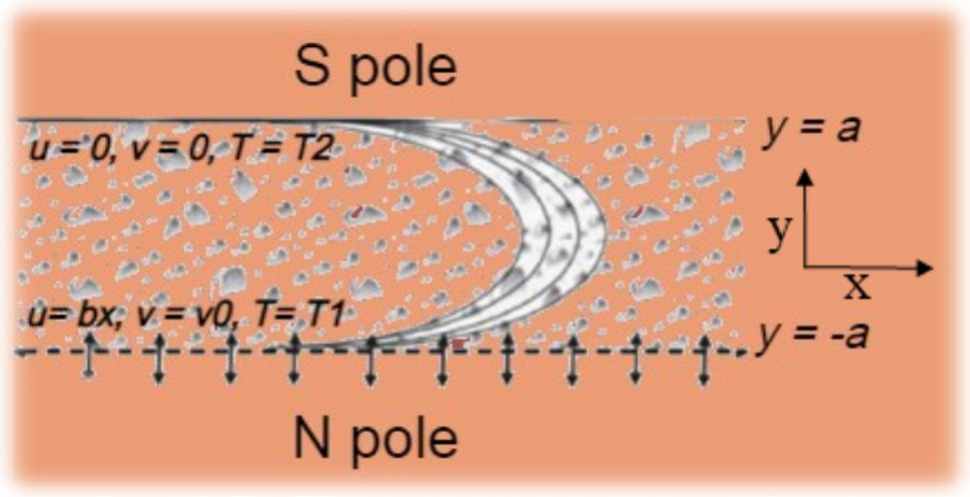
Geometry of a semi-porous channel.

In this study, we investigate a nanofluid's steady flow and heat transfer characteristics in a semi-porous channel. The geometry of the channel and its boundary conditions are illustrated in Fig. 1. We consider the impact of a constant vertical magnetic field on the nanofluid's flow and heat transfer characteristics. Additionally, we consider the effects of thermal radiation and Joule heating on the temperature distribution within the channel. The channel consists of a hot lower plate and a cold upper plate, with the nanofluid flowing steadily between them. This configuration allows us to investigate the heat transfer behaviors of the nanofluid in the presence of various forces. By considering the impact of various factors on temperature distribution, we can enhance our understanding of the underlying mechanisms of heat transfer in nanofluids.

The study presented the continuity equation and Navier–Stokes to evaluate the system's heat transfer^32^.

1 $$\frac{\partial u}{\partial x}+\frac{\partial v}{\partial y}=0$$

2 $${\rho }_{\mathrm{nf}}\left(u\frac{\partial u}{\partial x}+v\frac{\partial u}{\partial y}\right)=-\frac{\partial p}{\partial x}+{\mu }_{nf}\left(\frac{{\partial }^{2}u}{\partial {y}^{2}}\right)-{\sigma }_{nf}{B}_{0}{ }^{2}u$$

3 $${\rho }_{nf}\left(u\frac{\partial v}{\partial x}+v\frac{\partial v}{\partial y}\right)=-\frac{\partial p}{\partial y}+{\mu }_{nf}\left(\frac{{\partial }^{2}v}{\partial {x}^{2}}\right)$$

4 $${\left(\rho {C}_{p}\right)}_{nf}\left(u\frac{\partial T}{\partial x}+v\frac{\partial T}{\partial y}\right)={k}_{nf}\left(\frac{{\partial }^{2}T}{\partial {y}^{2}}\right)+{\sigma }_{nf}{B}_{0}{ }^{2}{u}^{2}-\frac{\partial {q}_{r}}{\partial y},$$

The text explains the consideration of radiation heat flux in the form of the Rosseland approximation, where the Stefan-Boltzmann constant ${(\sigma }_{e})$ and the mean absorption coefficient ${(\beta }_{R})$ play a key role. Specifically, the radiation heat flux (${q}_{r}$) is given by the expression ${q}_{r}=-\frac{4{\sigma }_{e}}{3{\beta }_{R}}\frac{\partial {T}^{4}}{\partial y}$^33^.

For this study, it is assumed that the differences in fluid-phase temperatures within the flow are small enough for ${(T}^{4})$ to be expressed as a linear function of temperature. This is achieved through the expansion of $({T}^{4})$ in a Taylor series around the temperature ${(T}_{C})$, with higher-order terms being neglected. The resulting expression is ${T}^{4}\cong 4{T}_{c}^{3}T-3{T}_{c}^{4}$. In other words, the study accounts for radiation heat flux within the flow by utilizing the Rosseland approximation. This involves considering the Stefan-Boltzmann constant and the mean absorption coefficient in calculating the radiation heat flux. Additionally, the Taylor series expansion is used to simplify the expression of $({T}^{4})$ and account for small differences in fluid-phase temperatures within the flow. By applying these methods, the study aims to provide a more accurate understanding of heat transfer behavior within the system. The effective properties of the nanofluid play a crucial role in the study of its behavior within the flow. Specifically, we are interested in the effective density, effective heat capacity, and electrical conductivity of the nanofluid, which are defined as follows^32^:

5 $${\rho }_{nf}=(1-\varphi ){\rho }_{f}+\varphi {\rho }_{p}$$

6 $${\left(\rho {C}_{p}\right)}_{nf}=(1-\varphi ){\left(\rho {C}_{p}\right)}_{f}+\varphi {\left(\rho {C}_{p}\right)}_{p}$$

7 $$\frac{{\sigma }_{nf}}{{\sigma }_{f}}=1+\frac{3\left(\frac{{\sigma }_{p}}{{\sigma }_{f}}-1\right)\varphi }{\left(\frac{{\sigma }_{p}}{{\sigma }_{f}}+2\right)-\left(\frac{{\sigma }_{p}}{{\sigma }_{f}}-1\right)\varphi }$$

The effective density is the ratio of the mass flow rate to the volumetric flow rate of the nanofluid. It considers the presence of the nanoparticles and their effect on the overall density of the fluid. The effective heat capacity represents the amount of heat required to increase the temperature of the nanofluid by a certain amount. It accounts for both the base fluid's heat capacity and the nanoparticles' contributions. The electrical conductivity of the nanofluid represents its ability to conduct electrical current. It is affected by various factors, including the concentration and size of the nanoparticles, as well as the properties of the base fluid.

By defining these effective properties, we can better understand the behavior of the nanofluid within the flow and how it responds to different forces such as magnetic fields, radiation, and Joule heating. This, in turn, can lead to more accurate modeling and prediction of heat transfer characteristics in a wide range of applications. Also, the KKL (Koo-Kleinstreuer-Li) correlation has been employed to determine the viscosity of the nanofluid in this study^3,34^. The KKL correlation is a commonly utilized method for estimating the viscosity of nanofluids. It is based on the theory that the viscosity of a nanofluid is dependent on various factors, such as the size and shape of the nanoparticles that make up the fluid. The correlation considers these factors and provides an estimation of the fluid's viscosity, which is a crucial parameter in determining the flow characteristics in the system. By employing the KKL correlation method, this study aims to determine the viscosity of the nanofluid under investigation more accurately. This, in turn, will lead to a better understanding of the flow properties of the fluid and the heat transfer characteristics of the system as a whole^34^:

8 $$\begin{array}{cc}{\mu }_{eff}& ={\mu }_{\text{static }}+{\mu }_{\text{Brownian }}={\mu }_{\text{static }}+\frac{{k}_{\text{Brownian }}}{{k}_{f}}\times \frac{{\mu }_{f}}{{\mathrm{Pr}}_{f}}\\ {k}_{\text{Brownian }}& =5\times {10}^{4}\varphi {\rho }_{f}{c}_{p,f}\sqrt{\frac{{\kappa }_{b}T}{{\rho }_{p}{d}_{p}}}{g}^{{{\prime}}}\left(T,\varphi ,{d}_{p}\right)\\ {g}^{\mathrm{^{\prime}}}\left(T,\varphi ,{d}_{p}\right)& =\left({a}_{1}+{a}_{2}\mathrm{ln}\left({d}_{p}\right)+{a}_{3}\mathrm{ln}(\varphi )+{a}_{4}\mathrm{ln}(\varphi )\mathrm{ln}\left({d}_{p}\right)+{a}_{5}\mathrm{ln}{\left({d}_{p}\right)}^{2}\right)\mathrm{ln}(T)\\ & +\left({a}_{6}+{a}_{7}\mathrm{ln}\left({d}_{p}\right)+{a}_{8}\mathrm{ln}(\varphi )+{a}_{9}\mathrm{ln}(\varphi )\mathrm{ln}\left({d}_{p}\right)+{a}_{10}\mathrm{ln}{\left({d}_{p}\right)}^{2}\right).\end{array}$$

The related coefficient and properties of Cuo-water nanofluid are presented in Table 1. Maxwell model and HamiltonCrosser model for irregular particle geometries by introducing a shape factor can be expressed as^35,36^:

**Table 1:** Thermo physical properties^32^.

	$\rho \left(\frac{\mathrm{kg}}{{\mathrm{m}}^{3}}\right)$	${c}_{p} \left(\frac{\mathrm{J}}{\mathrm{kg}.^\circ \mathrm{K}}\right)$	$k \left(\frac{\mathrm{W}}{\mathrm{m}.^\circ \mathrm{K}}\right)$	$\sigma {(\Omega \mathrm{ m})}^{-1}$
CuO	6500	540	18	0.05
Pure water	997.1	4179	0.613	10^–10^

9 $$\frac{{k}_{nf}}{{k}_{f}}=\frac{{k}_{p}+(m+1){k}_{f}-(m+1)\varphi \left({k}_{f}-{k}_{p}\right)}{{k}_{p}+(m+1){k}_{f}+\varphi \left({k}_{f}-{k}_{p}\right)}$$

The given equation employs ${k}_{p}$ and ${k}_{f}$ to respectively represent the conductivities of the particle material and the base fluid. The shape factor is denoted by "$m$" in the equation. Ref.^32^ presents distinct values of shape factors (3, 3.7, 4.8, and 5.7) for different shapes of nanoparticles (Spherical, Brick, Cylinder, and Platelet), respectively. This article discusses the use of copper oxide nanoparticles (CuO) as a material in the coating of boats, as well as their implementation as a base fluid in nanofluids. The study specifically focuses on the effects of water as a base fluid with CuO nanoparticles on the value of the Nusselt number. Through the research conducted, it was found that CuO nanoparticles are effective in preventing the growth of E. coli bacteria in suspension in low concentrations. CuO nanoparticles also possess magnetic properties, making them suitable for use in ferrofluids. Implementing CuO nanoparticles in various applications highlights their potential as a versatile material capable of being used in different settings and fields.

In the following, we consider B.C.s as:

10 $$\begin{array}{cc}& u=bx,v=-{v}_{0},T={T}_{1} \text{ at }y=-a\\ & u=0,v=0,T={T}_{2} \text{ at }y=+a\end{array}$$

the value of $b$ is less than zero for a channel with shrinking walls and greater than zero for a channel with stretching walls. The method of similarity transformation has been used to obtain ODEs. In addition, non-dimensional parameters are introduced to help with the analysis^32^.

11 $$\eta =\frac{y}{a},u=bx{f}^{{{\prime}}}(\eta ),v=-abf(\eta ),\theta =\frac{T-{T}_{1}}{{T}_{1}-{T}_{2}}$$

The final Eqs. (12–13) can be obtained using the earlier transformation in Eq. (11).

12 $${f}^{iv}-H{a}^{2}\frac{{A}_{5}}{{A}_{2}}{f}^{{{\prime \prime}}}-R\frac{{A}_{1}}{{A}_{2}}\left({f}^{{{\prime}}}{f}^{{{\prime \prime}}}-f{f}^{{{\prime \prime \prime}}}\right)=0$$

13 $$\left(1+\frac{4}{3{A}_{4}}Rd\right){\theta }^{{{\prime \prime}}}+\mathrm{Pr}\frac{{A}_{3}}{{A}_{4}}f{\theta }^{{{\prime}}}+H{a}^{2}Ec\frac{\mathrm{Pr}}{R}\frac{{A}_{5}}{{A}_{4}}{f}^{{{\prime}}2}=0,$$

Equations (8) and (9) contain various parameters represented by different symbols, such as $R$ for Reynolds number, $Ec$ for Eckert number, $Ha$ for Hartmann number, $Rd$ for radiation parameter, and $Pr$ for Prandtl number. The specified values for $A1- A5$ are also included in the equations^32^.

14 $$\begin{array}{cc}R& =\frac{{a}^{2}b}{{U}_{f}}, Ha={B}_{0}a\sqrt{\frac{{\sigma }_{f}}{{\mu }_{f}}}, Rd=4{\sigma }_{e}{T}_{c}^{3}/\left({\beta }_{R}{k}_{f}\right),\\ Ec& =\frac{{\rho }_{f}(bx{)}^{2}}{{\left(\rho {C}_{p}\right)}_{f}\Delta T},\mathrm{ Pr}=\frac{{a}^{2}b{\left(\rho {C}_{p}\right)}_{f}}{{k}_{f}},\\ {A}_{1}& =\frac{{\rho }_{nf}}{{\rho }_{f}}, {A}_{2}=\frac{{\mu }_{nf}}{{\mu }_{f}},{ A}_{3}=\frac{{\left(\rho {C}_{p}\right)}_{nf}}{{\left(\rho {C}_{p}\right)}_{f}},{ A}_{4}=\frac{{k}_{nf}}{{k}_{f}},{ A}_{5}=\frac{{\sigma }_{nf}}{{\sigma }_{f}}.\end{array}$$

Also, the B.C.s of ODEs are as follows:

15 $$\begin{array}{cc}& {f}^{{{\prime}}}(-1)=1,{f}^{{{\prime}}}(1)=0,\theta (-1)=1,\\ & f(-1)=\lambda =\frac{{v}_{0}}{ab},f(1)=0,\theta (1)=0.\end{array}$$

## Methodology

This study utilizes both Akbari-Ganji's and Homotopy perturbation methods to solve a specific problem. These applied techniques provide accurate solutions and offer several advantages over conventional methods. For instance, Akbari-Ganji's method is highly effective in transforming nonlinear problems into linear ones. This process enables well-established linear analytic techniques, simplifying the computation process. On the other hand, the Homotopy Perturbation Method is a powerful tool for solving problems with complex nonlinearities. It provides an iterative series solution that converges rapidly to the exact solution.

### The basic idea of HPM

We assume Eq. (16) as a base function and Eq. (17) as BC^37^:

16 $$A\left(u\right)-f\left(r\right)=0$$

17 $$B\left(u,\frac{\partial u}{\partial n}\right)=0$$

And $A(u)$ is described:

18 $$A\left(u\right)=L\left(u\right)+N\left(u\right)$$

HPM principle is written

19 $$H\left(\nu ,P\right)=\left(1-P\right)\left[L\left(\nu \right)-L\left({u}_{0}\right)\right]+P\left[N\left(v\right)-f\left(r\right)\right]=0$$

Or

20 $$H\left(v,P\right)=L\left(v\right)-L\left({u}_{0}\right)+PL\left({u}_{0}\right)+p\left[N\left(v\right)-f\left(r\right)\right]=0$$

where

21 $$v\left(r,P\right):\Omega \times \left[\mathrm{0,1}\right]\to R$$

After following Eqs. (19) and (20):

22 $$H\left(v,0\right)=L\left(v\right)-L\left({u}_{0}\right)=0, \,\,\,\,\,H\left(v,1\right)=A\left(v\right)-f\left(r\right)=0$$

The parameter $P$, which belongs to the interval $[\mathrm{0,1}]$, is an embedding parameter, ${u}_{0}$ represents initial approximation that fulfills the B.C. A change in parameter $P$ from 0 to 1 causes the transformation of $v(r,p)$ from u_0_ to ${u}_{r}$. In this context, $v$ is considered a function.

23 $$v={v}_{0}+p\times {v}_{1}+{P}^{2}\times {v}_{2}+\dots$$

And the best approximation is

24 $$u=\underset{P\to 1}{lim}v={v}_{0}+{v}_{1}+{v}_{2}$$

### The basic idea of AGM

To implement analytical methods for both linear and nonlinear differential equations, B.C.s and I.C.s are necessary. Confirming the differential equations in the following form is commonplace^38^:

25 $${p}_{k}:f\left(u,{u}^{{{\prime}}},{u}^{{{\prime \prime}}},\dots ,{u}^{(n)}\right)=0, u=u(x)$$

So, the order ($n$) and nonlinearity of the differential equation $\left({\mathrm{P}}_{\mathrm{k}}\right)$ can be determined, along with the form of boundary conditions, which take the following shape:

26 $$\begin{array}{c}u(0)={u}_{0},{u}^{{{\prime}}}(0)={u}_{1},\dots ,{u}^{(m-1)}(0)={u}_{m-1}\\ u(L)={u}_{{L}_{0}},{u}^{{{\prime}}}(L)={u}_{{L}_{1}},\dots ,{u}^{(m-1)}(L)={u}_{{L}_{m-1}}\end{array}$$

The subsequent stage in the solution of the problem using AGM is to utilize polynomials that have constant coefficients, like the following:

27 $$f\left(\eta \right)=\sum_{k=0}^{n} {a}_{k}{e}^{-0.8k\eta }={a}_{0}+{a}_{1}{e}^{-0.8\eta }+{a}_{2}{e}^{-1.6\eta }+{a}_{3}{e}^{-2.4\eta }+{a}_{4}{e}^{-3.2\eta }+{a}_{5}{e}^{-4\eta }+{a}_{6}{e}^{-4.8\eta }+{a}_{7}{e}^{-5.6\eta }$$

28 $$g(\eta )=\sum_{k=0}^{n} {b}_{k}{e}^{-0.8k\eta }={b}_{0}+{b}_{1}{e}^{-0.8\eta }+{b}_{2}{e}^{-1.6\eta }+{b}_{3}{e}^{-2.4\eta }+{b}_{4}{e}^{-3.2\eta }+{b}_{5}{e}^{-4\eta }+{b}_{6}{e}^{-4.8\eta }$$

So when $$\eta =0$$:

29 $$\begin{array}{cc}& u(0)={a}_{0}={u}_{0}\\ & {u}^{{{\prime}}}(0)={a}_{1}={u}_{1}\\ & {u}^{{{\prime \prime}}}(0)={a}_{2}={u}_{2}\end{array}$$

And $$\eta =L$$ :

30 $$\begin{array}{cc}& u(L)={a}_{0}+{a}_{1}L+{a}_{2}{L}^{2}+\dots +{a}_{n}{L}^{n}={u}_{{L}_{0}}\\ & {u}^{{{\prime}}}(L)={a}_{1}+2{a}_{2}L+3{a}_{3}{L}^{2}+\dots +n{a}_{n}{L}^{n-1}={u}_{{L}_{1}}\\ & {u}^{{{\prime \prime}}}(L)=2{a}_{2}+6{a}_{3}L+12{a}_{4}{L}^{2}+\dots +n(n-1){a}_{n}{L}^{n-2}={u}_{{L}_{n-1}}\end{array}$$

## Results and discussion

**Figure 2:**
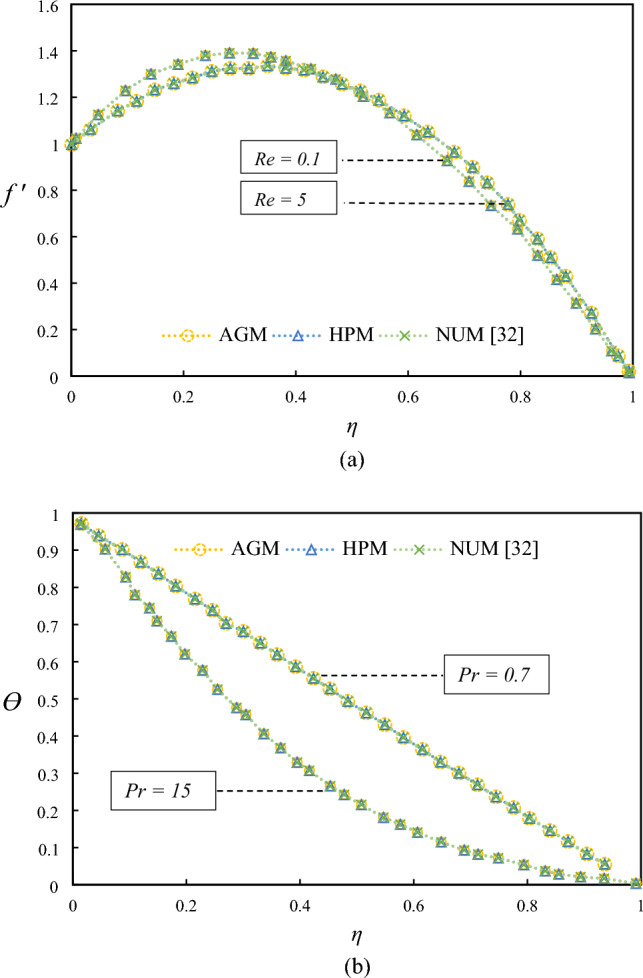
Examining differences in temperature and velocity profiles for varying values of $Re$ (**a**) and $Pr$ (**b**) when $R = 0.5, M = 1, \lambda = 0.5, Kr = 0.5$, by comparing AGM, HPM, and numerical method^32^.

The research focused on analyzing the effect of thermal radiation on the flow and heat transfer of a Magnetohydrodynamic (MHD) CuO-water nanofluid in a semi-porous channel with a stretching wall. The energy equation takes into account the influences of Joule heating. ODEs were obtained through a similarity transformation and were solved using AGM and HPM methods. Verifying the presented results with those of the previous work showed good accuracy, as depicted in Fig. 2 and Tables 2 and 3.

**Table 2:** Numerical values for comparison between different methods $Pr=0.7, 1, 2 , R=2.0, Ec=0.5, \lambda = 0.5, Kr=0.5.$

$Pr$	${f}{^\prime}_{HPM}$	${f}{^\prime}_{AGM}$	${f}{^\prime}_{HAM[39]}$	${f}{^\prime}_{NUM[32]}$
0.7	− 0.3176381	− 0.3178415	− 0.3178090	− 0.3176102
1.0	− 0.5151890	− 0.5153835	− 0.5153790	− 0.5152071
2.0	− 0.0536619	− 0.0539982	− 0.0539821	− 0.0537362

**Table 3:** Comparison between the skin friction coefficient and local Nusselt number results and Refs results.

$\phi$	$Cf{Re}_{x}^{1/2}=-\frac{1}{(1-\phi {)}^{2.5}}{f}^{{\prime}{\prime}}(0)$			$N{u}_{x}{Re}_{x}^{-1/2}=-\frac{{k}_{nf}}{{k}_{f}}{\theta }{\prime}\left(0\right)$		
	Cu-water			Cu-water		
	Mabood et al.^40^	Ghadikolae et al.^41^	Present study	Mabood et al.^40^	Ghadikolae et al.^41^	Present study
0	1.6871	1.6872	1.6871	1.7148	1.7148	1.7148
0.02	1.7622	1.7623	1.7621	1.8021	1.8021	1.8021
0.04	2.1245	2.1245	2.1244	1.8196	1.8196	1.8196

Refs.^40,41^ for different values of nanoparticle volume fraction when $Pr = 6.2$.

Figure 2 showed differences in temperature and velocity profiles for varying values of $Re$ (a), $Pr$ (b) when $R = 0.5, M = 1, \lambda = 0.5$*,* and $Kr = 0.5$, by comparing AGM, HPM, and Numerical method^32^ and Table 2 presents a comparison of the results obtained from the AGM and HPM proposed in this study with those obtained from a numerical method presented in^32^ and HAM in^39^. The comparison is conducted for various parameter values. By providing a numerical comparison, we can demonstrate the validity of the proposed methods and their ability to produce accurate results compared to other established methods. Table 3 compares the skin friction coefficient and local Nusselt number obtained from this study for different values of nanoparticle volume fraction when $Pr = 6.2$, with the results presented in Refs.^40,41^. The comparison demonstrates that the results obtained from this study are consistent with previous research studies, reinforcing our findings' validity. In addition, Table 4 shows the influence of nanoparticle shape on the local Nusselt number for various types of nanoparticles when $Pr = 6.2$. Through the analysis, it was found that platelet-shaped nanoparticles provide the maximum Nusselt number. Therefore, we have selected Platelet nanoparticles for further investigation, and this information will be useful in designing more efficient heat transfer systems. The results presented in Tables 1, 2, 3 and 4 provide valuable insights into the thermal conductivity of fluids and the utilization of nanoparticles as enhancers for heat transfer.

**Table 4:** Nusselt number for various shapes of the nanoparticles when $Pr = 6.2$.

R	Platelet	Cylinder	Brick	Spherical
1	1.815141623	1.790365832	1.757657289	1.735301472
1.5	3.259502784	3.247115981	3.230758836	3.219568394
2	3.911240356	3.905464827	3.897842716	3.892635983
2.5	4.241593872	4.240017726	4.237986042	4.236621824

**Figure 3:**
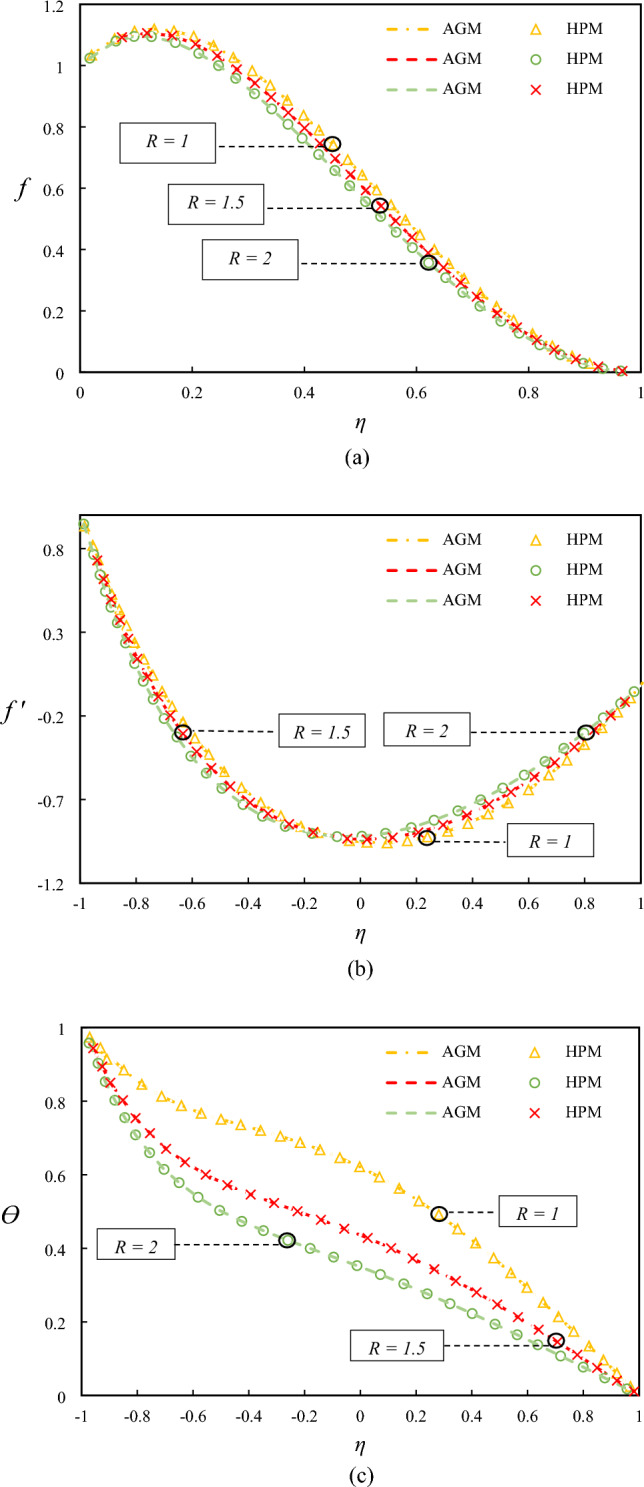
Examining differences in temperature and velocity profiles for varying values of $Re$ by comparing AGM, HPM.

In Fig. 3, the impact of Reynolds number on the velocity and temperature profiles is shown when $Rd = 0.5, Ha = 1, Ec = 0.5, \lambda = 1, \varphi = 0.04,$ and $Pr = 6.2$. The observed decrease in the temperature profiles and the vertical velocity with an increase in Reynolds number can be attributed to increased fluid turbulence and mixing, causing a reduction in heat transfer efficiency. The decrease in the horizontal velocity near the down wall can be explained by the presence of the solid boundary, which causes a damping effect on the fluid motion. On the other hand, the opposite trend observed in the proximity of the upper wall can be attributed to the orientation of the fluid flow, which is influenced by the buoyancy force imposed by the temperature gradient.

**Figure 4:**
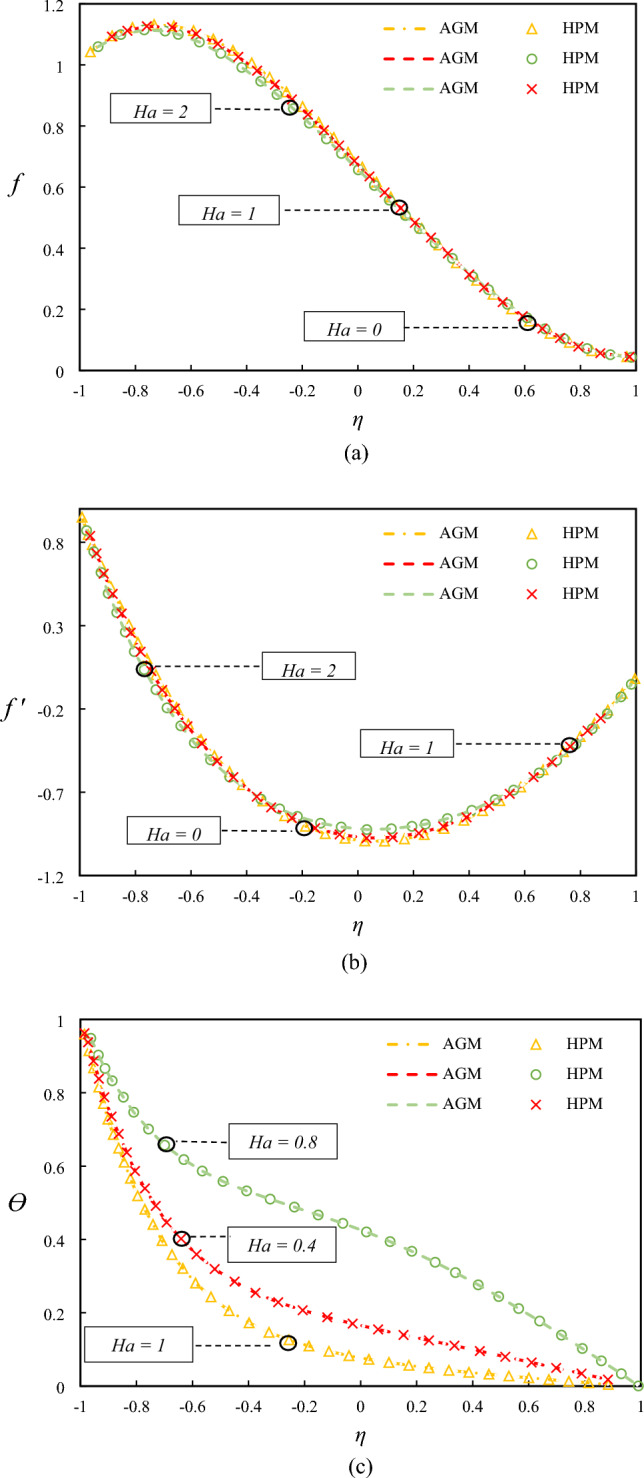
Examining differences in temperature and velocity profiles for varying values of $Ha$ by comparing AGM, HPM.

Figure 4 presents the impact of Hartmann number on the temperature and velocity profiles. The observed enhancement in the temperature profile with an increase in the Hartmann number can be attributed to increased heat transfer efficiency due to the applied magnetic field, which suppresses the mixing of the fluid and thus enhances the thermal boundary layer. The reduction in the velocity with increasing conductivity can be explained by the Lorentz force, which acts against the fluid motion and thus reduces the fluid velocity.

**Figure 5:**
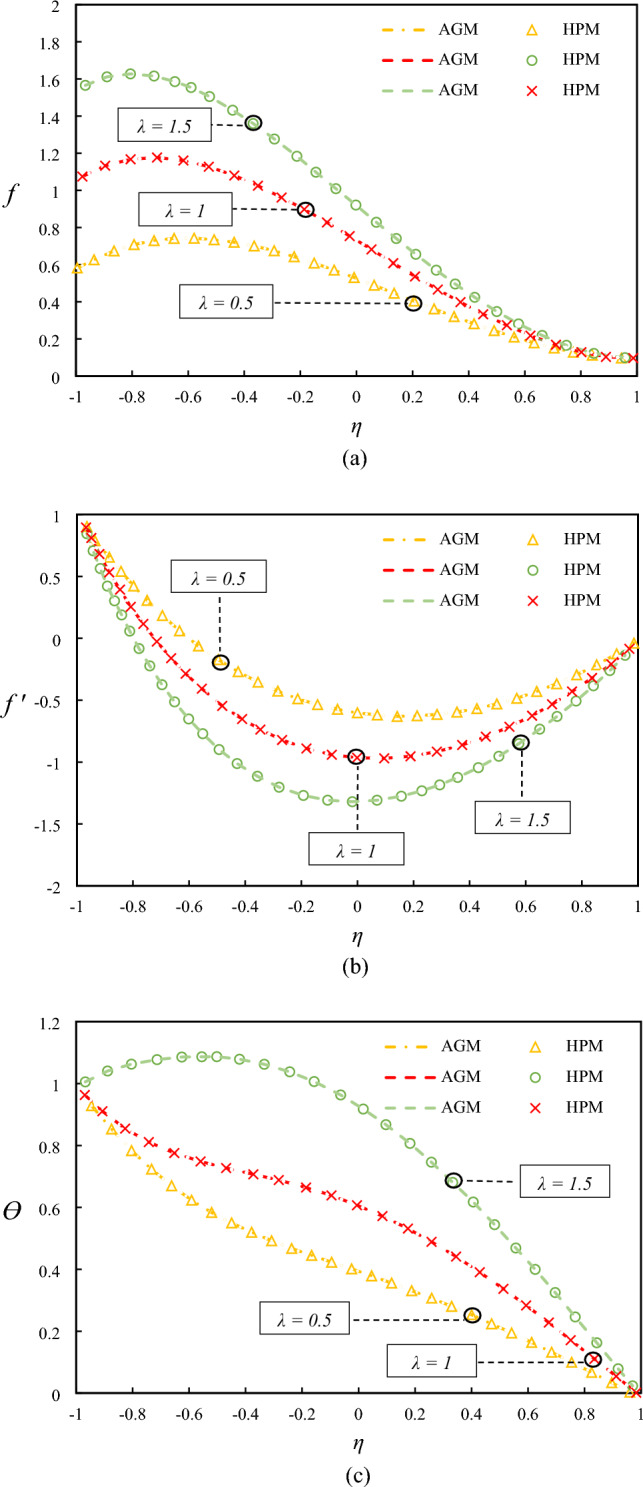
Examining differences in temperature and velocity profiles for varying values of $\lambda$ by comparing AGM, HPM.

Figure 5 shows the impact of the suction parameter on the temperature and velocity profiles. The increased mixing and turbulence in the fluid flow enhances the temperature profile and the vertical velocity with an increase in the suction parameter can be explained by the increased mixing and turbulence, which enhances the convective heat transfer. The reduction in the horizontal velocity can be attributed to the suction effect, which draws fluid towards the wall and thus reduces the flow velocity in the central region. Additionally, the shift in the min point of velocity to the lower wall can be attributed to the effect of suction on the flow structure, which alters the flow patterns near the wall. These physical justifications provide a deeper understanding of the observed trends and enhance the discussion on the impact of different parameters on fluid flow and heat transfer.

**Figure 6:**
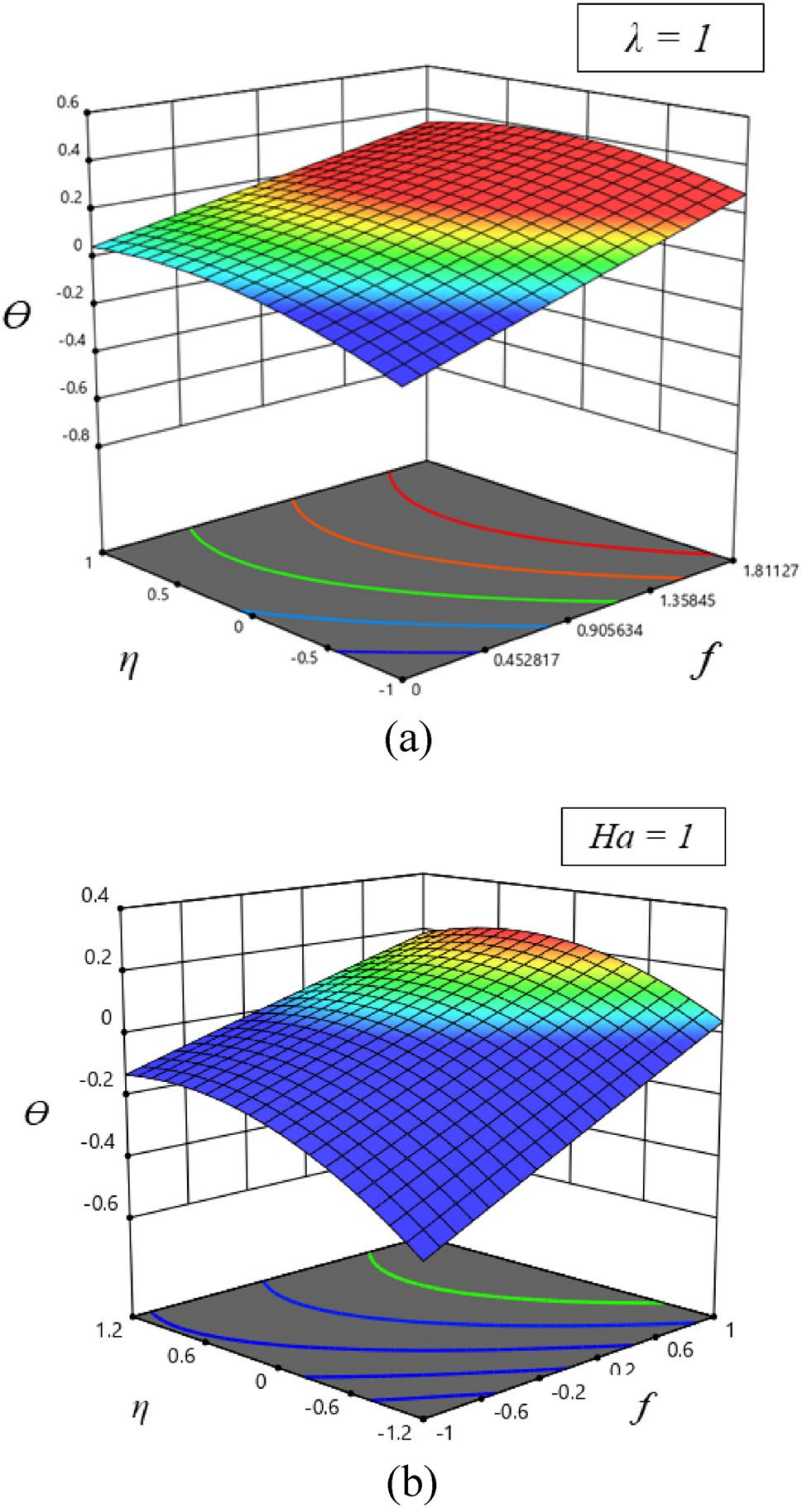
3D surface of velocity (**b**) and temperature (**a**) profiles.

Also, to better understand the results, Fig. 6 showed 3D surface plots of the model for different values of $f(\eta )$ on the temperature and velocity when $\gamma = 0.5$ and $Ha = 1$. According to these results, temperature increases when velocity increases in different values of $\gamma$. The results presented here can serve as a basis for further research and development, particularly in understanding the effects of individual parameters on the behavior of these models. These findings offer valuable insights into the behavior of complex systems of differential equations and provide a strong foundation for future research in related areas.

## Conclusion

After conducting a detailed study on the flow and heat transfer of Magnetohydrodynamic (MHD) CuO-water nanofluid in a semi-porous channel with a stretching wall, we can conclude that the proposed methods (AGM and HPM) are highly effective and accurate in predicting the behavior of fluids. The analyses demonstrated interesting results on the influence of several factors, such as nanofluid volume fraction, Hartmann number, nanoparticle shape, Eckert number, suction parameter, and radiation parameter, on the temperature and velocity profiles. Specifically, we found that the temperature profile decreased with increasing nanofluid volume fraction, while Platelet-shaped nanoparticles provided the highest Nusselt number. Additionally, we observed that the applied magnetic field and suction parameter were the key drivers for enhancing the heat transfer efficiency of the fluid. Furthermore, the presented comparison with previous work and numerical methods reinforces the validity of the results.

In summary, the research findings highlight the importance of investigating different parameters and physical processes in accurately predicting the behavior of fluids. In future investigations, we recommend further exploration into the impact of particle size, fluid flow rates, and other numerical methods. Additionally, exploring the effect of other physical phenomena, such as chemical reactions or phase changes, on flow and heat transfer can provide deeper insights into the thermal conductivity of fluids. By continuing to analyze the behavior of fluids, we can develop better heat transfer and flow systems for various industrial and technological applications.

## Data Availability

Data will be shared by corresponding author on request.

## List of symbols

$${B}_{0}$$: Magnetic induction
$$Rd$$: Radiation parameter
$$Ha$$: Hartmann number
${C}_{p}$: Specific heat capacity
$$\mathrm{Pr}$$: Prandtl number
$${q}_{r}$$: Thermal radiation
$$T$$: Fluid temperature
$v,u$: Vertical and horizontal velocities

## Greek symbols

$$\sigma$$: Electrical conductivity
$\eta$: Similarity independent variable
$\varphi$: Volume fraction of nanofluid
$$\sigma$$: Surface tension
${\sigma }_{e}$: Stefan–Boltzmann constant
$$\alpha$$: Thermal diffusivity
${\beta }_{R}$: Mean absorption coefficient
$$\mu$$: Dynamic viscosity

## Subscripts

$$s$$: Solid particles
$$f$$: Base fluid
$$nf$$: Nanofluid
C: Solutal quantity
$$T$$: Thermal quantity
$$\infty$$: For $$\eta \to \infty$$
0: At $$\eta \to 0$$
